# Endoscopic-Assisted Transoral Thyroglossal Cyst Resection

**DOI:** 10.3389/fendo.2021.774174

**Published:** 2022-02-18

**Authors:** Shanwen Chen, Dong Wang, Jianxin Qiu, Yehai Liu, Yi Zhao

**Affiliations:** Department of Otorhinolaryngology-Head and Neck Surgery, The First Affiliated Hospital of Anhui Medical University, Hefei, China

**Keywords:** endoscopic surgery, thyroglossal cyst, transoral procedures, outcome, cosmetic (plastic) surgery

## Abstract

Sistrunk procedure is the standard method for thyroglossal duct cyst resection. While this procedure is successful and safe, it results in postoperative scars on the front of neck. We propose a total transoral technique without external incision that starts with careful separation of the floor of the mouth and genioglossus muscle followed by the exact localization of the cyst using methylene blue. Simultaneously, the hyoid bone connected to the cyst and tract was removed. Finally, routine hemostasis is conducted, and the operative cavity is closed. All patients who received this operation in our department recovered successfully without experiencing severe intraoperative or postoperative complications.

## Introduction

Thyroglossal duct cyst (TGDC) is one of the most commonly observed neck masses in clinical medicine. It is characterized by an active, painless mass in the midline or slightly to one side of the neck, usually located below the hyoid bone (about 75% of patients), and can move along with the tongue. This cyst develops embryologically when the thyroid primordium descends from the base of the tongue to its typical location in front of the trachea. If the tract does not degenerate, clinical cysts may develop (1).

Sistrunk procedure is the widely accepted surgical treatment for TGDC and is similar to open thyroid surgery. This procedure entails the removal of the central part of hyoid bone and thyroid remnants. However, the procedure leaves a roughly 5 cm scar in front of the neck.

Kim et al. (2) were the first to report a successful transoral TGDC resection. No other teams have reported the clinical application of this method. Considering different details, our goal is to share our experience with this approach. Informed consent and ethical review were obtained (IRB number: PJ2021-03-22).

## Methods

**Figure 1:**
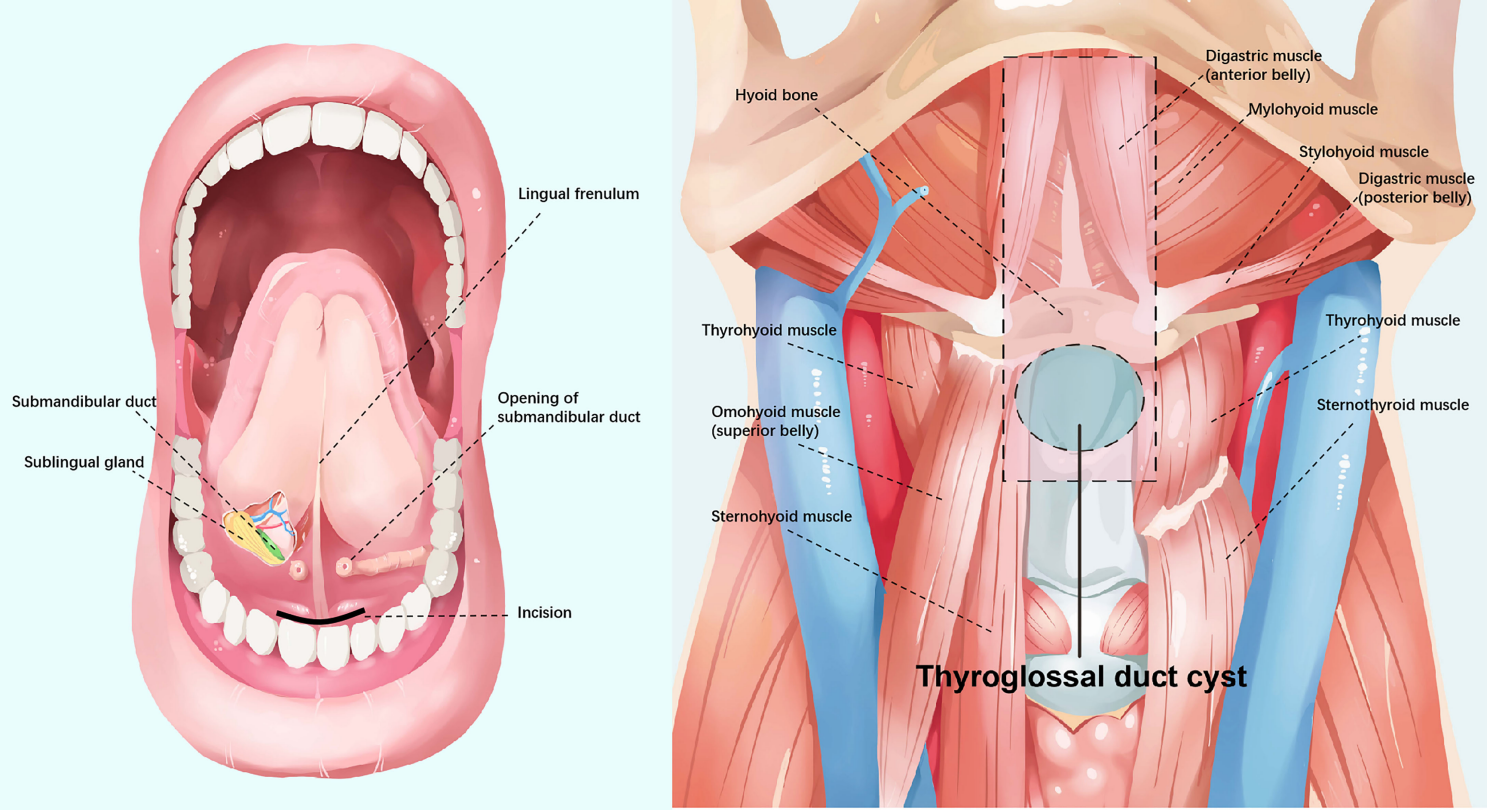
Illustration of anatomy.

**Figure 2:**
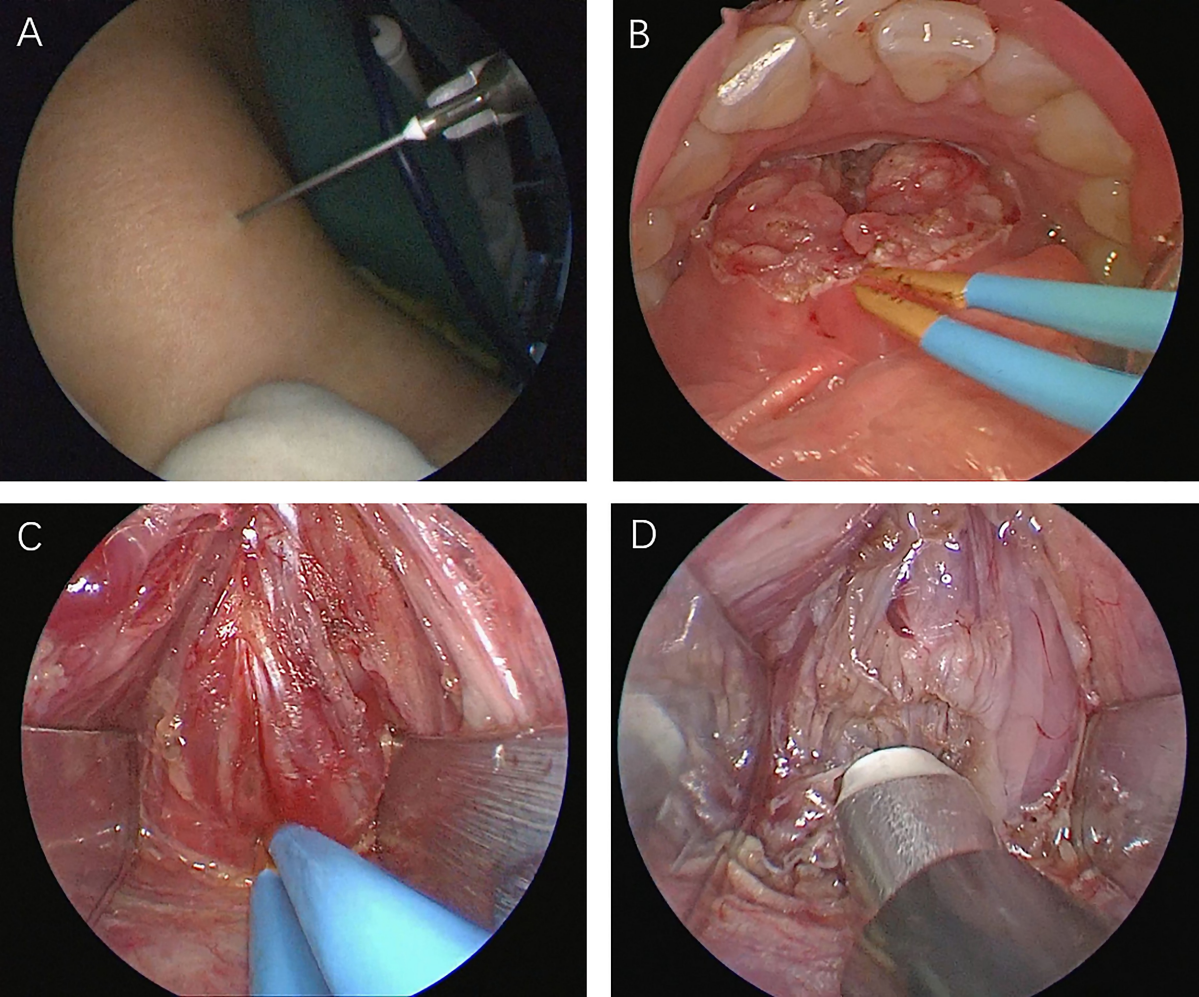
**(A)** Methylene blue injection. **(B)** Transverse frenotomy incision. **(C)** The genioglossus muscles were retracted bilaterally. **(D)** Transection of suprahyoid muscles.

**Figure 3:**
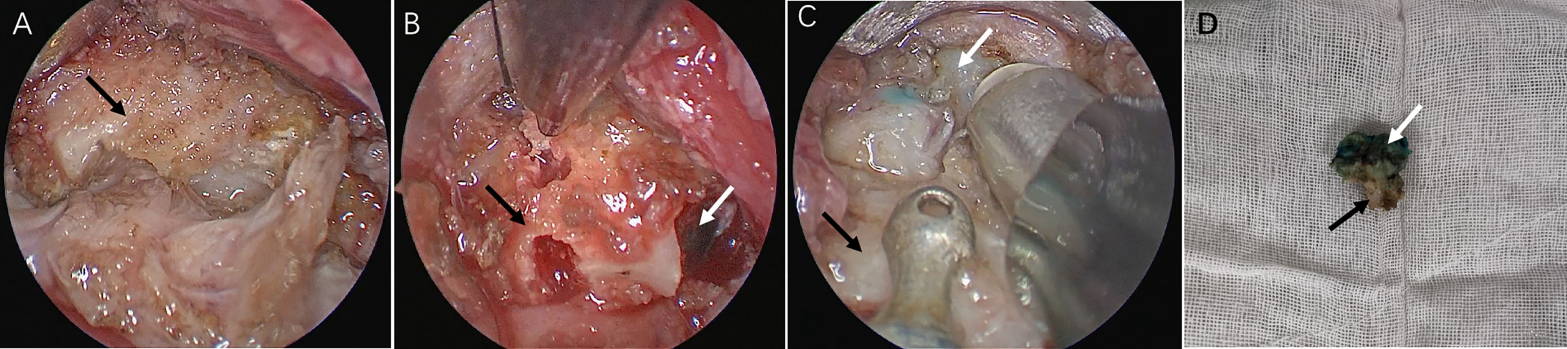
**(A)** Identification of hyoid bone (black arrow). **(B)** Identification of TGDC (white arrow). **(C)** Dissection of the cyst. **(D)** Specimen.

We describe this procedure using the example of a 24-year-old woman who required it for cosmetic purposes. Therefore, we decided to perform a scarless procedure. **Figure 1** illustrates relevant anatomical diagrams. The proposed technique was conducted under general anesthesia and oral endotracheal intubation. To maintain oral operating space, a unilateral oral distractor was employed. Subsequently, methylene blue was injected into the cyst from the outside (**Figure 2A**). At the oral lingual frenulum, a transverse incision of about 2 cm in length was made (**Figure 2B**). The soft tissue of the mouth floor was meticulously dissected, and genioglossus muscles with marked morphological characteristics were identified, separated, and retracted bilaterally (**Figure 2C**). Using a 4 mm rigid nasal endoscope, we longitudinally separated and transected suprahyoid muscles (**Figure 2D**). Then the hyoid bone was located and identified (**Figure 3A**). The hyoid bone’s body was then cut using a Kerrison rongeur (**Figure 3B**). Following this step, it was often found that the partially blue-stained cyst remained in the operation cavity. We employed a cryogenic plasma knife to transect the infrahyoid muscles at a distance of 0.5 cm from the hyoid bone. By pulling the hyoid bone upward using forceps, the cyst was completely exposed. Using a plasma knife, the tissue surrounding the cyst was meticulously dissected, and the hyoid bone was pulled out (**Figure 3C**). The hyoid bone and cyst were removed together (**Figure 3D**). After sufficient hemostasis, the operative cavity was flushed with distilled water. A drainage tube was inserted from the mouth floor and fixed under the chin. Finally, the oral mucosa was sutured with a 4-0 suture and bandaged with external neck pressure (**Video 1, Supporting Information**).

The drain was removed when drainage volume was less than 5 mL per 24 h. After operation, no special dietary restrictions were found, and patients were given saline to gargle and maintain their mouth clean. Patients were discharged two days following extubation unless there were special circumstances and were required to comply with regular outpatient follow-up.

## Results

**Figure 4:**
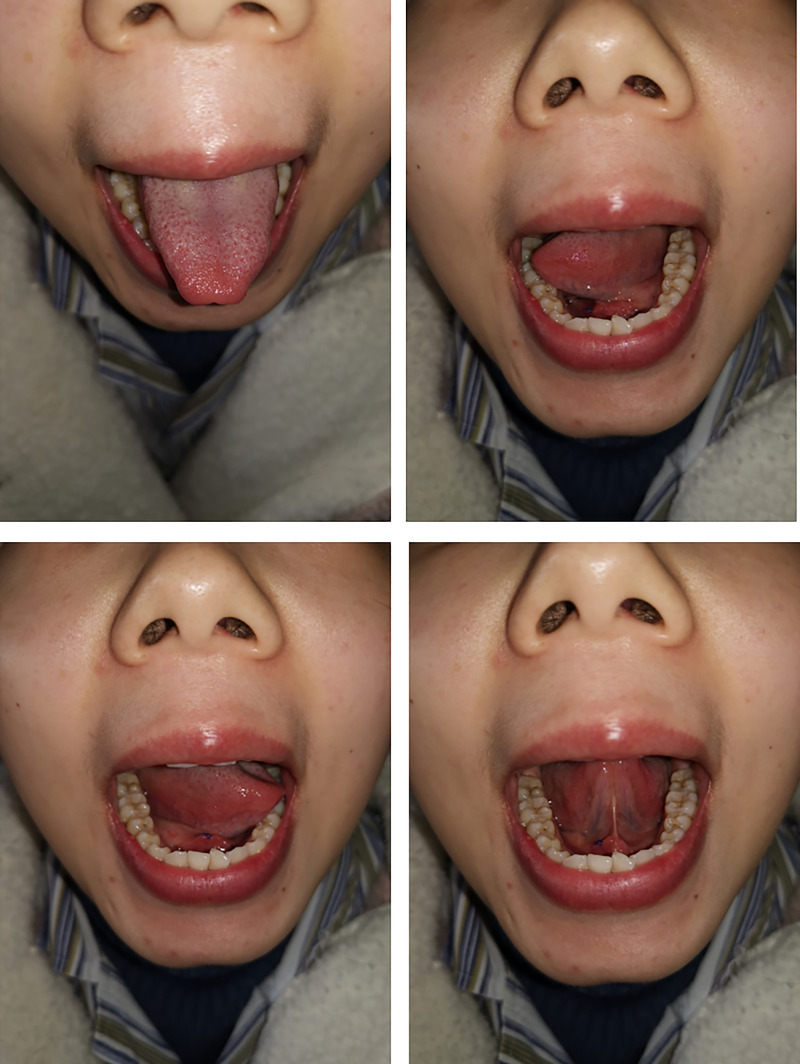
Tongue movement after operation.

**Figure 5:**
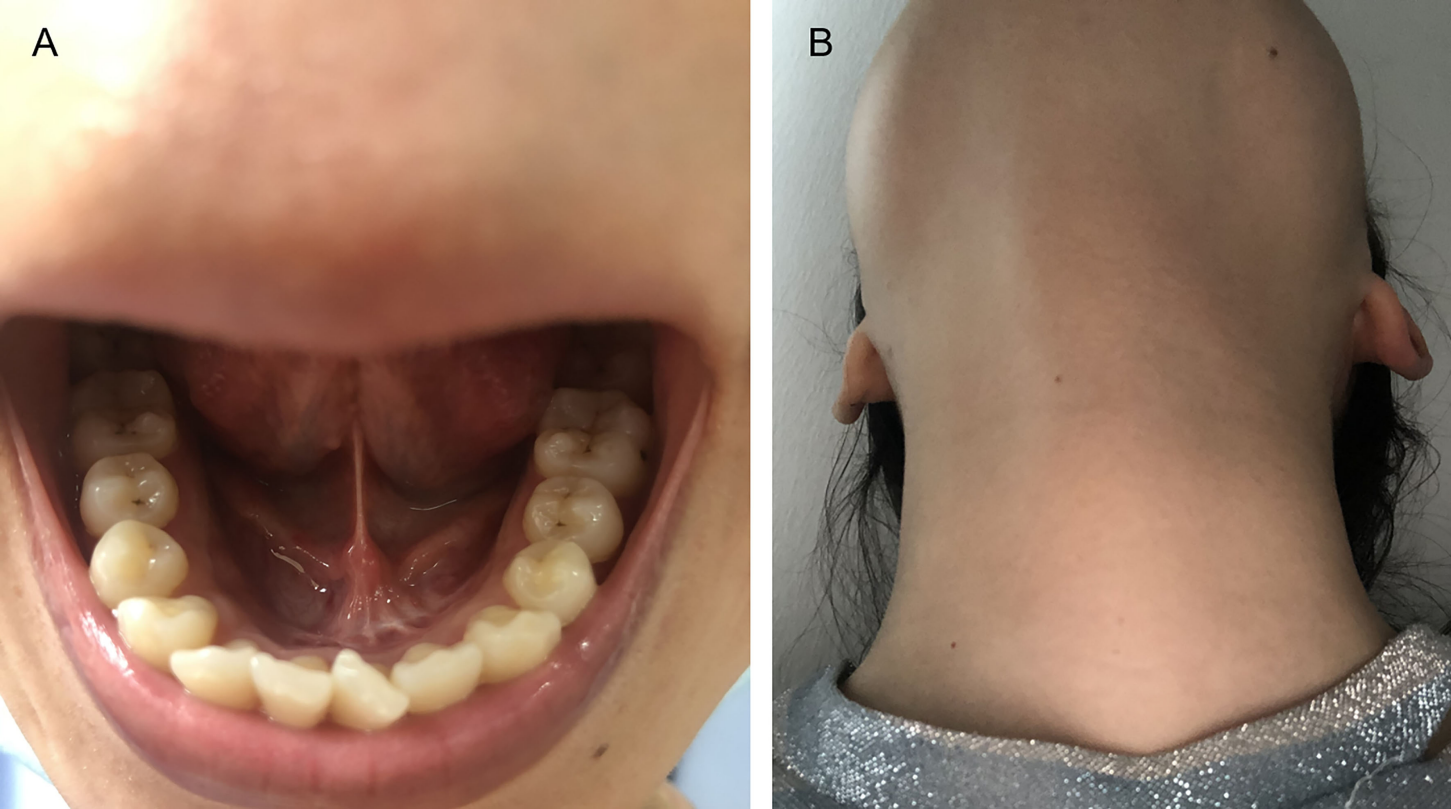
**(A)** Incision at 6-month follow-up. **(B)** Appearance at 6-month follow-up.

Our technique was successful in five patients. The characteristics of patients and operations are listed in **Table 1**. During at least a half-year follow-up, no recurrences or peri/postoperative complications were observed, and all patients achieved excellent cosmetic outcomes with their necks (**Figures 4** and **5**).

**Table 1:** Patient demographics and outcomes for undergoing transoral approach for TGDC.

Patient	Age,y	Sex	Lesion	Operative	Bleeding	Drainage	Complication
			size (cm)	time (min)	volume (ml)	volume (ml)	
1	43	Female	1.5×1.2×0.5	180	10	10	None
2	42	Female	2.0×2.0×1.0	190	10	35	None
3	24	Female	1.5×1.0×0.6	210	15	10	None
4	34	Female	2.0×1.0×0.5	165	15	20	None
5	22	Female	2.0×1.4×0.4	130	5	30	None

TGDC, thyroglossal duct cyst.

## Discussion

After diagnosis with a thyroglossal duct cyst, patients tended to seek surgical treatment with an otolaryngologist. A standard Sistrunk procedure is often adopted to resect the lesion. However, growing esthetic concerns motivate patients to seek cosmetic treatment for their unsightly scar in the neck. While the auxiliary use of local drugs and the improvement of surgical hardware can indeed reduce the obvious degree of postoperative scarring, they can also introduce complications such as pruritus and local pain.

Currently, researchers have focused on hidden scars or scarless cosmetic surgery. Cai et al. (3) used endoscope-assisted surgery to successfully resect TGDC *via* a small submental incision, but scarring remains inevitable. One study published by Qu et al. (4) presented a study in which they successfully executed the breast approach on 13 patients, but one had an infection, one developed skin bruising, and another had subcutaneous fluid. Anuwong et al. (5) also completed this operation with the bilateral areola approach. Considering the author’s experience with endoscopic surgery, none of the 11 patients included in their study experienced postoperative complications, and the operation time was shorter. Paek et al. (6) successfully completed TGDC resection for two patients using the bilateral axillo-breast approach. However, this procedure requires more incisions. Kim et al. (7) and Lee et al. (8) selected the retroauricular approach, successfully removed TGDC with robot assistance, and the incision can be hidden, but the costs and hardware constraints obstruct wider accessibility. Han et al. (9) and Banuchi et al. (10) completed the operation by employing the transoral vestibular approach. The former adopts a single incision without insufflation, while the latter adopts the same three incisions with CO_2_ insufflation as the vestibular approach for thyroid. Both operations were completed successfully without complications. However, the single incision method of Han et al. (9) does not avoid the problem of narrow operation space. Asians have a relatively flat jaw, which may impede the use of this technology among other races. Compared with the above approaches, the distance of frenotomy incision is the shortest, and this approach conforms to the law of anatomy. The surgical path is positioned in the midline without important vessels and nerves and does not require extensive tissue dissection and CO_2_ insufflation, which avoids possible complications of hypercapnia and numbness in the operation area. However, it should be noted that limitations are still present. The frenotomy incision may bring the risk of incision infection; sublingual edema may cause airway obstruction; narrow operation space significantly increases the difficulty of this procedure, requiring more patience and time. Our operation time was between two and four hours, while the time reported by Woo et al. (11) was about one hour. Similarly, the operation time of Anuwong et al. (5) is also shorter compared with Qu et al. (4) We believe that this technique can be executed more efficiently as experience grows.

The tongue must be retracted upward to expose the mouth floor and the frenum. After recognizing Wharton’s duct orifice, we made a frenotomy incision closer to the mandible. Then, blunt separation was used for tissue dissection. In so doing, risk of Wharton’s duct injury can be avoided (12). The next issue is the limited operating space, implying why we used 4 mm nasal endoscope. Based on our experience, it is critical to precisely locate the cyst when using the transoral approach. As a result, we used methylene blue for intraoperative localization. The next step requires the surgeon to have extensive experience with endoscopic procedures to avoid cyst rupture because the rupture would lead to blue staining in the operation field, making boundary identification is difficult.

At present, there remain a few reports on endoscopic TGDC surgery, and there are insufficient materials to define indications and contraindications of this operation. In our study, the patients had strong cosmetic intentions and had no history of neck surgery, neck radiotherapy, thyroglossal cyst infection, or thyroglossal fistula. In the above literature, most specimens are 2-3 cm, with the largest specimen of 6 cm in the study of Woo et al. (11) There is no consensus on lesion size limitation. In our method, syringe can be utilized to extract part of the capsule, which may overcome the sample size limitation, but this must be confirmed by follow-up research.

## Conclusion

TGDC removal using a frenotomy incision is an alternative to conventional procedure with a safe, effective, cosmetic, and well-tolerated outcome. However, because the sample size was relatively small, additional research on indications, complications, and recurrences is still required.

## Data Availability Statement

The raw data supporting the conclusions of this article will be made available by the authors, without undue reservation.

## Ethics Statement

The studies involving human participants were reviewed and approved by Ethics Committee of the First Affiliated Hospital of Anhui Medical University. The patients/participants provided their written informed consent to participate in this study.

## Author Contributions

Concept and design: All authors. Acquisition, analysis, or interpretation of data: All authors. Drafting of the manuscript: SC. Critical revision of the manuscript for important intellectual content: All authors. Statistical analysis: SC. Administrative, technical, or material support: All authors. Supervision: YZ. All authors contributed to the article and approved the submitted version.

## Conflict of Interest

The authors declare that the research was conducted in the absence of any commercial or financial relationships that could be construed as a potential conflict of interest.

## Publisher’s Note

All claims expressed in this article are solely those of the authors and do not necessarily represent those of their affiliated organizations, or those of the publisher, the editors and the reviewers. Any product that may be evaluated in this article, or claim that may be made by its manufacturer, is not guaranteed or endorsed by the publisher.
